# Antioxidant Effect Assessment and Trans Epithelial Analysis of New Hempseed Protein Hydrolysates

**DOI:** 10.3390/antiox12051099

**Published:** 2023-05-15

**Authors:** Guillermo Santos-Sánchez, Gilda Aiello, Fabrizio Rivardo, Martina Bartolomei, Carlotta Bollati, Anna Arnoldi, Ivan Cruz-Chamorro, Carmen Lammi

**Affiliations:** 1 Instituto de Biomedicina de Sevilla, IBiS, Hospital Universitario Virgen del Rocío, CSIC, Universidad de Sevilla, 41013 Seville, Spain; gsantos-ibis@us.es; 2 Departamento de Bioquímica Médica y Biología Molecular e Inmunología, Facultad de Medicina, Universidad de Sevilla, 41009 Seville, Spain; 3 Department of Pharmaceutical Sciences, University of Milan, via Mangiagalli 25, 20133 Milan, Italy; martina.bartolomei@unimi.it (M.B.); carlotta.bollati@unimi.it (C.B.); anna.arnoldi@unimi.it (A.A.); 4 Department of Human Science and Quality of Life Promotion, Telematic University San Raffaele, 00166 Rome, Italy; gilda.aiello@uniroma5.it; 5 A. Costantino & C. Spa, Via Francesco Romana 11-15, 10083 Favria, Italy; frivardo@acostantino.com

**Keywords:** hempseed, bioactive peptides, antioxidant, functional foods, Caco-2 cells, bioavailability

## Abstract

Hempseed (*Cannabis sativa*) is one of the most promising sources of plant proteins. It contains approximately 24% (*w*/*w*) protein, and edestin accounts for approximately 60–80% (*w*/*w*) of its total proteins. In a framework of research aimed at fostering the proteins recovered from the press cake by-products generated after the extraction of hempseed oil, two hempseed protein hydrolysates (HH1 and HH2) were produced at an industrial level using a mixture of different enzymes from *Aspergillus niger*, *Aspergillus oryzae*, and *Bacillus licheniformis* for different times (5 h and 18 h). Using a combination of different direct antioxidant tests (DPPH, TEAC, FRAP, and ORAC assays, respectively), it has been demonstrated that HHs exert potent, direct antioxidant activity. A crucial feature of bioactive peptides is their intestinal bioavailability; for this reason, in order to solve this peculiar issue, the ability of HH peptides to be transported by differentiated human intestinal Caco-2 cells has been evaluated. Notably, by using mass spectrometry analysis (HPLC Chip ESI-MS/MS), the stable peptides transported by intestinal cells have been identified, and dedicated experiments confirmed that the trans-epithelial transported HH peptide mixtures retain their antioxidant activity, suggesting that these hempseed hydrolysates may be considered sustainable antioxidant ingredients to be exploited for further application, i.e., nutraceutical and/or food industries.

## 1. Introduction

In recent years, the use of plant proteins in the food, nutraceutical, and cosmetic industries has consolidated. Besides their nutritional characteristics, these proteins have additional value when turned into bioactive hydrolysates, which show potential beneficial activities for human health [1,2]. The enzyme-catalyzed treatment is the most common procedure used to prepare vegetal protein hydrolysates. In fact, the proteolytic reaction breaks down the primary sequence, generating a mixture of peptides with potential lipid-lowering [3,4,5], antioxidant [6], antihypertensive [7], anti-inflammatory [8], anticancer [9], and antimicrobial [9] properties, among others. The combination of these potential properties and their capability to reach the site of action make these molecules highly appealing.

The antioxidant activity is the most investigated property; in fact, peptides contribute to the antioxidant capacity of cells and to the maintenance of the biological health of tissues. Some of the mechanisms of action involved in the antioxidant activity of peptides are the inactivation of reactive oxygen species (ROS), the scavenging of free radicals, reductions in hydroperoxides, and the inhibition of linoleic acid oxidation [10,11]. Structural features of protein hydrolysates, such as amino acid composition, molecular weight, and peptide size, influence antioxidant activities. For instance, higher proportions of hydrophobic amino acids (aa) are present in protein hydrolysates, which possess strong free radical scavenging properties [12]. Low molecular weight peptides (<3 kDa) are the most active since they can interact with radicals efficiently and easily exert their antioxidant capacity through the in vivo intestinal barrier. Peptides can cross the intestinal barrier intact and carry out their bioactivity at the tissue level [13].

Hempseed (*Cannabis sativa*) is one of the most promising sources of plant protein. It contains approximately 24% (*w*/*w*) protein, and edestin accounts for approximately 60–80% (*w*/*w*) of its total proteins [14,15,16]. Hempseed proteins have been hydrolyzed into peptides with different properties [17], including antihypertensive [18,19,20], α-glucosidase inhibitory [21], hypocholesterolemic [22,23], anti-inflammatory [24], and antioxidant [25,26]. The present study, considering the current interest in antioxidant peptides obtained from food sources, aims to promote the bioactivities of two hempseed protein hydrolysates (HHs) by focusing the interest on the identification and characterization of bioavailable antioxidant peptides. The two HHs were generated using a mixture of different enzymes from *Aspergillus niger*, *Aspergillus oryzae*, and *Bacillus licheniformis* for different times (5 and 18 h), in a framework of research aimed at fostering the proteins recovered from the press cake by-products generated after the extraction of hempseed oil. Other hydrolysates of hempseeds have been generated using one or two enzymes, with pepsin [18,27,28], Alcalase [29,30], pancreatin [31], and Flavourzyme [32] being the most used. Nevertheless, the use of a proper combination of enzymes could result in the generation of new bioactive peptides with potential bioactivity. In more detail, the first objective of the work was the in vitro evaluation of the antioxidant activities of the HHs using the most widely used antioxidant assay (1,1-diphenyl-2-picrylhydrazyl (DPPH) radical, Trolox equivalent antioxidant capacity (TEAC), ferric reducing antioxidant power (FRAP), and the oxygen radical absorbance capacity (ORAC) assays). A crucial feature of peptides is their intestinal bioavailability; for this reason, the second objective of this study was the HHs intestinal trans-epithelial transport evaluation. To achieve this goal, differentiated Caco-2 cells were used as a natural sieve of the bioavailable peptide species. In addition, using the same cellular system, HH antioxidant effects were assessed by measuring both the intracellular reactive oxygen species (ROS) and nitric oxide (NO) levels, respectively. Finally, the third objective of this work was to evaluate the antioxidant activity of the absorbed peptides by performing FRAP and DPPH assays, respectively.

## 2. Materials and Methods

### 2.1. Hempseed Hydrolysates (HHs) Production

The hempseed hydrolysate 1 (HH1) and HH2 were obtained using two different cocktails of proteases in collaboration with A. Costantino & C. Spa. For HH1, digestive enzymes from *Aspergillus niger* (pectin lyase, polygalacturonase, and cellulase) and *Aspergillus oryzae* (oryzin and leucyl aminopeptidase) were used. For HH2, digestive enzymes from Aspergillus niger and *Bacillus licheniformis* (subtilisin) were employed. The time of hydrolysis was 5 h (HH1.5; HH2.5) or 18 h (HH1.18; HH2.18) (Table 1). In more detail, the 5 h and 18 h refer to two different hydrolysis times in which hempseed proteins were subjected. Therefore, in the former condition, the hydrolytic process of hempseed proteins is less extensive than in the latter conditions.

### 2.2. In Vitro Antioxidant Activities

#### 2.2.1. 1-Diphenyl-2-picrylhydrazyl Radical (DPPH) Assay

The DPPH solution (0.0125 mM in methanol, 45 μL) was added to 15 μL of the HHs at different concentrations (0.5–2.5 mg/mL). The reaction for scavenging DPPH radicals was performed in the dark at room temperature and the absorbance was measured at 520 nm after 30 min of incubation.

#### 2.2.2. Trolox Equivalent Antioxidant Capacity (TEAC)

TEAC assay was performed using the 2,2-azino-bis-(3-ethylbenzothiazoline-6-sulfonic acid) (ABTS) radical solution (Sigma-Aldrich, Milan, Italy). A total of 140 µL of this solution was mixed with 10 µL of sample at the final concentrations of 10, 25, and 50 µg/mL HHs. After 30 min of incubation at 30 °C, the ABTS radical content was quantified by a Synergy™ HT-multimode microplate reader (Biotek Instruments, Winooski, VT, USA) at 730 nm. The TEAC values were extrapolated by a Trolox (Sigma-Aldrich) standard curve.

#### 2.2.3. Ferric Reducing Antioxidant Power (FRAP) Assay

An amount of 10 µL of sample, at the final concentrations of 10, 25, and 50 µg/mL HHs, was mixed with 140 µL FRAP solution (0.83 mM TPTZ and 1.66 mM FeCl_3_ × 6H_2_O in 0.25 M acetate buffer, pH 3.6). After 30 min of incubation at 37 °C, the absorbance at 595 nm was measured with the Synergy reader (Biotek). The values were extrapolated by a Trolox (Sigma-Aldrich) standard curve.

#### 2.2.4. Oxygen Radical Absorbance Capacity (ORAC) Assay

An amount of 25 µL of sample, at the final concentrations of 10, 25, and 50 µg/mL HHs, was added to 50 µL of sodium fluorescein (2.93 µg/µL) (Sigma-Aldrich) and incubated for 15 min at 37 °C. Then, 25 µL azo2,20-azobis(2-methylpropionamidine) dihydrochloride (AAPH, 60.84 mM, Sigma-Aldrich) was added to generate peroxyl radicals. Thus, every 5 min, over 2 h, decay of fluorescein at its maximum emission of 528 nm was measured using the Synergy reader (Biotek). The area under the curve (AUC) was calculated using a Trolox (Sigma-Aldrich) calibration curve.

### 2.3. Mass Spectrometry Analysis (HPLC Chip ESI-MS/MS)

Both HH1 and HH2 were filtered using a 3 kDa cut-off. The flow-through from type 1 and 2 and the basolateral samples derived from the trans-epithelial transport experiment were dried using the speed-vacuum system and then dissolved in 50 μL of a solution of 99% water and 1% ACN containing 0.1% formic acid. A total of 2 μL of each sample was injected into a nano-chromatographic system, HPLC-Chip (Agilent Palo Alto, CA, USA). Each sample was loaded onto a 40 nL enrichment column (Zorbax 300SB-C18, Agilent Technologies, Santa Clara, CA, USA, 5 µm pore size) and separated onto a 43 mm × 75 µm analytical column (Zorbax 300SB-C18, 5 µm pore size). The separations were carried out in gradient mode at a flow rate of 500 nL/min. The elution solvent A was 95% water, 5% ACN, and 0.1% formic acid; solvent B was 5% water, 95% ACN, and 0.1% formic acid. The nano pump gradient program was as follows: 5% solvent B (0 min), 50% solvent B (0–50 min), 95% solvent B (50–60 min), and back to 5% for 10 min. Data acquisition occurred in positive ionization mode. The capillary voltage was −2000 V, with an endplate offset of −500 V. Full-scan mass spectra were acquired in the mass range from *m*/*z* 300 to 2000 Da. LC-MS/MS analysis was performed in data-dependent acquisition AutoMS(n) mode. In order to increase the number of identified peptides, three technical replicates (LC-MS/MS runs) were run for each hydrolysate. The MS/MS data were analyzed by the Spectrum Mill Proteomics Workbench (Rev B.04.00, Agilent), consulting the *Cannabis sativa* database downloaded from UniProtKB/Swiss-Prot—ExPASy. A nonspecific enzyme was selected as the cutting enzyme. Peptide mass tolerance was set to 1.0 Da and the fragment mass tolerance to 0.8 Da. An autovalidation strategy for both the peptide and protein polishing modes was performed using a false discovery rate (FDR) cut-off ≤ 1.2%.

### 2.4. Cell Culture and Cell Monolayers Integrity Evaluation

Human intestinal Caco-2 cells (INSERM, Paris, France) were cultured in complete high-glucose DMEM medium (with L-glutamine, 10% fetal bovine serum, 100 U/mL penicillin, and 100 μg/mL streptomycin) with incubation at 37 °C under 5% CO_2_ atmosphere. Caco-2 cells were routinely sub-cultured at 50% density. For differentiation, Caco-2 cells (3.5 × 10^5^ cells/cm^2^ density) were seeded on polycarbonate filters, 12 mm diameter, 0.4 μm pore diameter (Transwell, Corning Inc., Lowell, MA, USA), and cultured according to a published protocol [33]. The trans-epithelial electrical resistance (TEER) of differentiated Caco-2 cells was estimated using the voltmeter apparatus Millicell (Millipore Co., Burlington, MA, USA), before and at the end of the absorption experiments. Differentiated Caco-2 cells were considered when the TEER value was more than 800 Ω/cm^2^.

### 2.5. 3-(4,5-Dimethylthiazol-2-yl)-2,5-diphenyltetrazolium Bromide (MTT) Assay

Amounts of 3 × 10^4^ Caco-2 cells/well were seeded in 96-well plates and treated with HHs (5 h and 18 h) at 0.1–2.5 mg/mL or vehicle (H_2_O) in complete high-glucose DMEM medium for 48 h at 37 °C under 5% CO_2_ atmosphere. Afterwards, the solvent was removed, and 100 μL/well of filtered 3-(4,5-dimethylthiazol-2-yl)-2,5-diphenyltetrazolium bromide (MTT) solution was added. After 2 h of incubation, the solution was aspirated and 100 μL/well of the lysis buffer (8 mM HCl + 0.5% NP-40 in DMSO) was added. After 10 min of slow shaking, the absorbance at 575 nm was read on the microplate reader Synergy H1 (Biotek).

### 2.6. Trans-Epithelial Transport of Hemp Hydrolysates

Before proceeding with the experiments, the cell monolayer integrity and differentiation were controlled by TEER measurement, as described above. HHs trans-epithelial passage was assayed in differentiated Caco-2 cells in transport buffer solution (137 mM NaCl, 5.36 mM KCl, 1.26 mM CaCl_2_, 1.1 mM MgCl_2_, and 5.5 mM glucose) according to previously described conditions [34]. To replicate the pH conditions existing in vivo in the small intestinal mucosa, the AP solutions were maintained at pH 6.0 (buffered with 10 mM morpholinoethane sulfonic acid), and the BL solutions were maintained at pH 7.4 (buffered with 10 mM N-2-hydroxyethylpiperazine-N-4-butanesulfonic acid). Cells were then washed twice (with 500 μL PBS w/Ca**^++^**, Mg**^++^**), and the absorption was assayed by loading the AP compartment with 1.0 mg/mL of HHs (5 h and 18 h) in the AP transport solution (500 μL) and the BL compartment with the BL transport solution (700 μL). The plate was incubated at 37 °C and the BL solutions were gathered after 2 h. BL and AP solutions collected at the end of the transport experiment were stored at −80 °C prior to analysis.

### 2.7. Fluorometric Intracellular ROS Assay

For cell preparation, 3 × 10^4^ Caco-2 cells/well were seeded on a black 96-well plate overnight in growth medium. The day after, the medium was removed and replaced with 50 μL/well of complete DMEM and 50 μL/well of the Master Reaction Mix and the cells were incubated at 5% CO_2_, 37 °C, for 1 h in the dark. Then, cells were treated with 10 μL of 11x HHs (5 and 18 h) at final concentrations of 0.5–2.5 mg/mL and incubated at 37 °C for 24 h in the dark. To induce ROS, cells were treated with 10 μL of H_2_O_2_ at a final concentration of 1.0 mM for 1 h a 37 °C in the dark, and fluorescence signals (ex./em. 490/525 nm) were recorded using a Synergy H1 microplate reader.

### 2.8. Nitric Oxide Level Evaluation on Caco-2 Cells

Caco-2 cells (1.5 × 10^5^/well) were seeded on 24-well plates. The next day, cells were treated for 24 h with HHs (5 and 18 h) at 0.5–2.5 mg/mL or vehicle (H_2_O) and incubated at 37 °C under a 5% CO_2_ atmosphere. After incubation, cells were stimulated with H_2_O_2_ (1.0 mM) or vehicle for 1 h; then, the cell culture media were collected and centrifuged at 13,000× *g* for 15 min to remove insoluble material. NO determination was carried out by Griess test. Briefly, 1.0 g of Griess reagent powder was solved in 25.0 mL of distilled H_2_O and 50.0 μL of the solution was incubated with 50.0 μL of the culture supernatants for 15 min at RT in the dark. The absorbance was measured at 540 nm using the Synergy H1 fluorescent plate reader from Biotek.

### 2.9. DPPH Assay in BL Solution

A total of 10 µL of BL solution was mixed with 30 µL of 1-diphenyl-2-picrylhydrazyl radical (DPPH) solution (0.0125 mM in methanol; Sigma-Aldrich). After 30 min of incubation at room temperature and in the dark, the DPPH radicals was quantified with the Synergy reader (Biotek) at 515/520 nm.

### 2.10. Trolox Equivalent Antioxidant Capacity (TEAC) in BL Solution

A total of 10 µL of BL solution was used to perform the TEAC assay, as described above.

### 2.11. Statistical Analysis

Data were shown as the mean ± standard deviation (s.d.). All the data sets were checked for normal distribution by D’Agostino and Pearson tests. Since they are all normally distributed with *p*-values < 0.05, the mixed-effects analysis and one-way ANOVA test, followed by multiple comparisons and Tukey’s test correction, were applied to identify the statistical difference. A difference with a *p*-value ≤ 0.05 was considered statistically significant. To graph and analyze data, GraphPad Prism 9 was used (GraphPad Software, San Diego, CA, USA).

## 3. Results

### 3.1. In Vitro Antioxidant Capacity

To test the possible antioxidant capacity exerted by HHs, several antioxidant assays were performed. Thus, it was evaluated by DPPH, TEAC, FRAP, and ORAC assays.

#### 3.1.1. HHs have DPPH Radical Scavenging Activity

**Figure 1:**
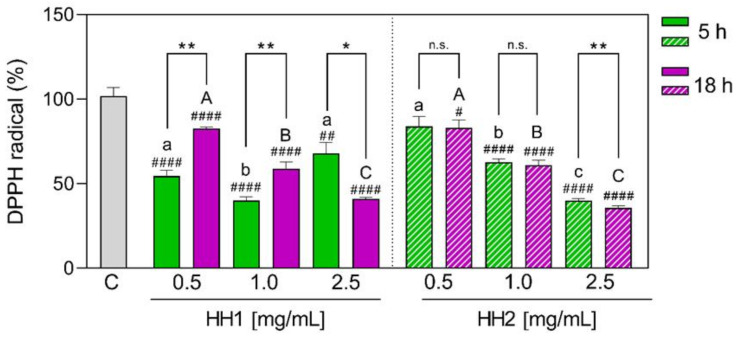
In vitro antioxidant power evaluation of the HHs by 2,2-diphenyl-1-picrylhydrazyl (DPPH) assay. C, control group; DPPH, 1-diphenyl-2-picrylhydrazyl radical; HH, hempseed hydrolysate. Data were shown as the mean ± standard deviation (*n* = 4). Mixed-effects analysis with multiple comparisons was applied. #, *p* ≤ 0.05; ##, *p* < 0.01; ####, *p* < 0.0001, with respect to the C group. *, *p* ≤ 0.05; **, *p* < 0.01; n.s., not significant. Different lower letters indicate the statistically significant difference (*p* ≤ 0.05) between the different concentrations of the HH obtained by 5 h of hydrolysis. Different upper letters indicate the statistically significant difference (*p* ≤ 0.05) between the different concentrations of the HH obtained by 18 h of hydrolysis.

As the first antioxidant activity screening, the DPPH radical scavenging activity was tested for the different concentrations of HHs. As Figure 1 shows, HH1.5 was capable of reducing the DPPH radical by 45.5 (*p* < 0.0001), 59.9 (*p* < 0.0001), and 32.0% (*p* = 0.009) at 0.5, 1.0, and 2.5 mg/mL, respectively. The concentration with the best results for HH1.5 was 1.0 mg/mL. In the same way, HH1.18 reduced the DPPH by 17.3 (*p* < 0.0001), 41.2 (*p* < 0.0001), and 59% (*p* < 0.0001) at increasing concentrations, finding significant differences between doses. When HH1.5 and HH1.18 were compared between them, significant differences were shown for each dose. Specifically, HH1.5 showed a higher capacity for scavenging the DPPH radical at 0.5 and 1.0 mg/mL in comparison to HH1.18, while HH1.18 showed a better activity at 2.5 mg/mL.

Focusing on HH2, HH2.5 and HH2.18 reduced the DPPH radical in a concentration-dependent way. Specifically, HH2.5 reduced the DPPH radical by 37.3 (*p* < 0.0001) and 60% (*p* < 0.0001) at 1.0 and 2.5 mg/mL, while HH2.18 diminished it by 16.9 (*p* = 0.015), 39.1 (*p* < 0.0001), and 64.3% (*p* < 0.0001), respectively. All the doses showed significant differences between them for each HH2. When HH2.5 and HH2.18 were compared, only the concentration of 2.5 mg/mL showed significant differences (*p* = 0.004).

#### 3.1.2. HHs Improve the Antioxidant Capacity by TEAC Radical Scavenging

**Figure 2:**
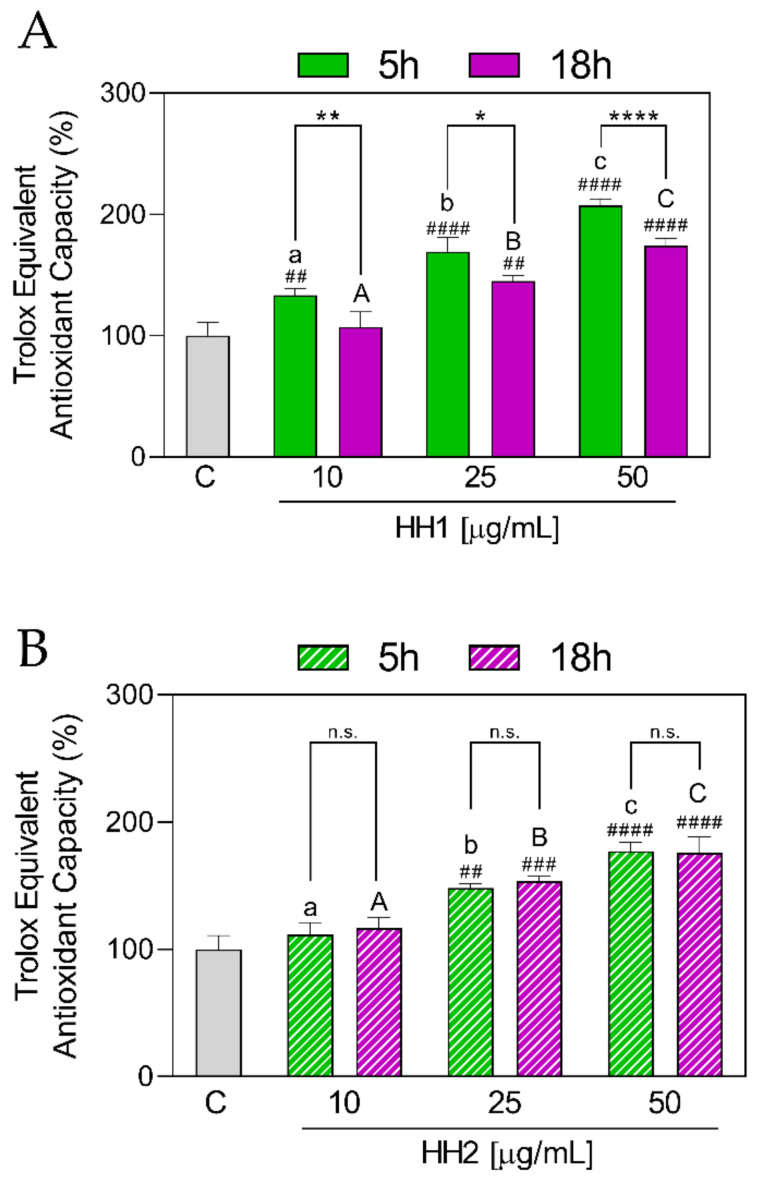
In vitro antioxidant power evaluation of the HHs by 2,2-azino-bis-(3-ethylbenzothiazoline-6-sulfonic acid) (ABTS) assay. C, control group; HH, hemp hydrolysate 1 (**A**) and 2 (**B**). Data were shown as the mean ± standard deviation (*n* = 10). Mixed-effects analysis with multiple comparisons was applied. ##, *p* < 0.01; ###, *p* < 0.001; ####, *p* < 0.0001, with respect to the C group. *, *p* ≤ 0.05; **, *p* < 0.01; ****, *p* < 0.0001; n.s., not significant. Different lower letters indicate the statistically significant difference (*p* ≤ 0.05) between the different concentrations of the HH obtained by 5 h of hydrolysis. Different upper letters indicate the statistically significant difference (*p* ≤ 0.05) between the different concentrations of the HH obtained by 18 h of hydrolysis.

As shown in Figure 2A, all the tested concentrations (10, 25, and 50 µg/mL) of HH1.5 were able to improve the TEAC, with the best effect observed at 50 µg/mL (10 µg/mL: 133 ± 5.70%, *p* = 0.0076; 25 µg/mL: 169 ± 11.80%, *p* < 0.0001; 50 µg/mL: 207 ± 5.28%, *p* < 0.0001). Moreover, a concentration-dependent response was observed.

On the other hand, although HH1.18 at 10 µg/mL did not exert a significant increase in TEAC values (107 ± 12.30%, *p* = 0.99 vs. C group), the greater concentrations showed an increase in TEAC values (25 µg/mL: 145 ± 4.61%, *p* = 0.0017; 50 µg/mL: 174 ± 5.56%, *p* < 0.0001), compared to the C group.

When HH1.5 and HH1.18 were compared between them, significant differences at each concentration were shown, with HH1.5 having the highest values (10 µg/mL, *p* = 0.007; 25 µg/mL, *p* = 0.026; 50 µg/mL, *p* < 0.0001).

Regarding HH2 (Figure 2B), it showed a similar scenario to HH1. Both hydrolysates increased TEAC at 25 µg/mL (HH2.5: 148 ± 3.50%, *p* = 0.0016; HH2.18: 154 ± 3.74%, *p* = 0.0008) and 50 µg/mL (HH2.5: 177 ± 6.93%, *p* < 0.0001; HH2.18: 50 µg/mL: 175 ± 12.70%, *p* < 0.0001). No differences were observed for both HHs at 10 µg/mL. Furthermore, the three concentrations tested (10, 25, and 50 µg/mL) for each condition (5 h and 18 h) showed significant differences between them.

Unlike HH1, when HH2.5 and HH2.18 at each concentration were compared with each other, no significant differences were observed (*p* < 0.05).

#### 3.1.3. HHs Improve the Ferric Reducing Antioxidant Power

**Figure 3:**
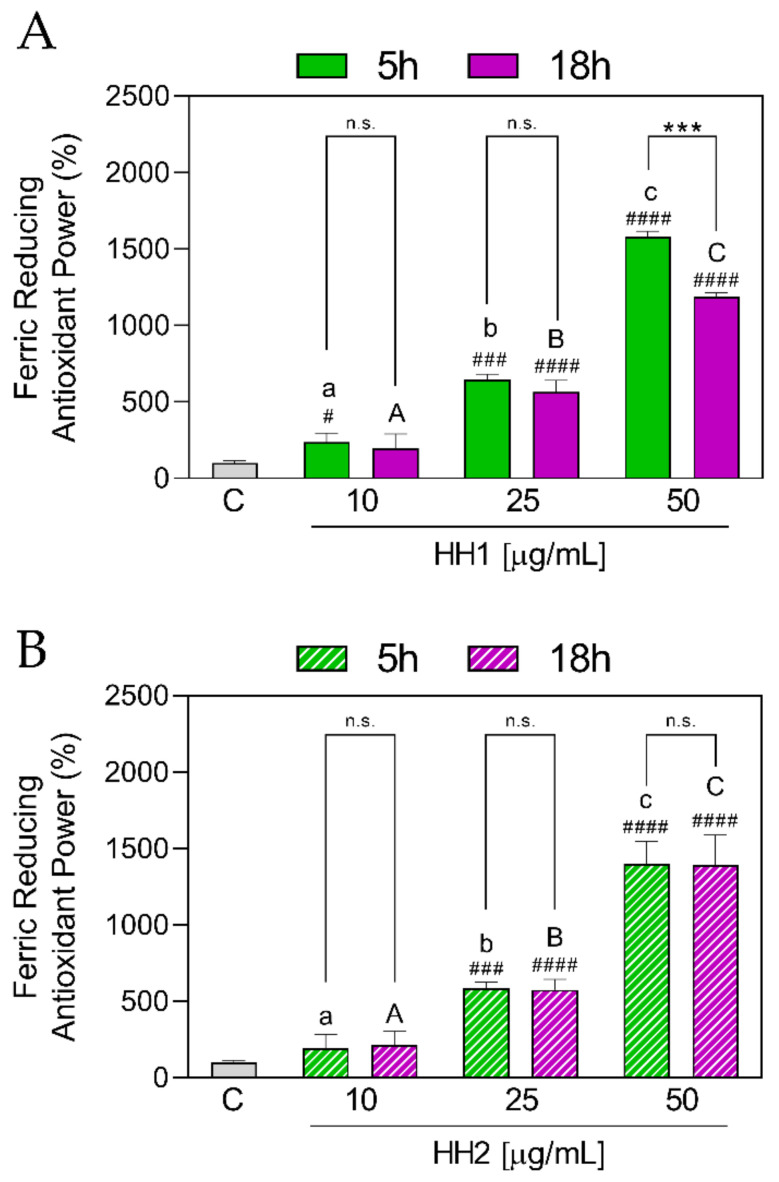
In vitro antioxidant power evaluation of the HHs by the ferric reducing antioxidant power (FRAP) assay. C, control group; HH, hemp hydrolysate 1 (**A**) and 2 (**B**). Data were shown as the mean ± standard deviation (*n* = 10). Mixed-effects analysis with multiple comparisons was applied. #, *p* < 0.05; ###, *p* < 0.001; ####, *p* < 0.0001, with respect to the C group. ***, *p* < 0.001; n.s., not significant. Different lower letters indicate the statistically significant difference (*p* ≤ 0.05) between the different concentrations of the HH obtained by 5 h of hydrolysis. Different upper letters indicate the statistically significant difference (*p* ≤ 0.05) between the different concentrations of the HH obtained by 18 h of hydrolysis.

The ferric reducing power of HHs was evaluated using the FRAP assay. The results showed that HH1.5 was capable of increasing the FRAP values in a concentration-dependent manner (10 µg/mL: 235 ± 60.70%, *p* = 0.0155; 25 µg/mL: 645 ± 36.80%, *p* = 0.0002; 50 µg/mL: 1578 ± 39.0%. *p* < 0.0001), with respect to the C group (Figure 3A). However, HH1.18 showed an increase in the FRAP values at 25 µg/mL (566 7 ± 73.40%, *p* < 0.0001) and 50 µg/mL (1186 ± 25.90%, *p* < 0.0001). In addition, differences between the concentrations were observed for each time condition (5 and 18 h). When HH1.5 and HH1.18 were compared between them, significant differences were observed between the two at the concentration of 50 µg/mL (*p* = 0.0002), with HH1.5 demonstrating the highest values. On the other hand, and in the same way that HH1, HH2.5, and HH2.18 were shown to increase FRAP levels at 25 (HH2.5: 589 ± 37.30%, *p* = 0.0002; HH2.18; 575 ± 69.60%, *p* = < 0.0001) and 50 (HH2.5: 1397 ± 150%, *p* < 0.0001; HH2.18; 1392 ± 195%, *p* < 0.0001) µg/mL (Figure 3B). A total of 10 µg/mL did not show significant differences in HH2 (*p* > 0.05).

Unlike HH1, HH2 did not show significant differences between HH2.5 and HH2.18 in any of the concentrations used (*p* > 0.05).

#### 3.1.4. HHs Improve Antioxidant Capacity by AAPH Radical Scavenging

**Figure 4:**
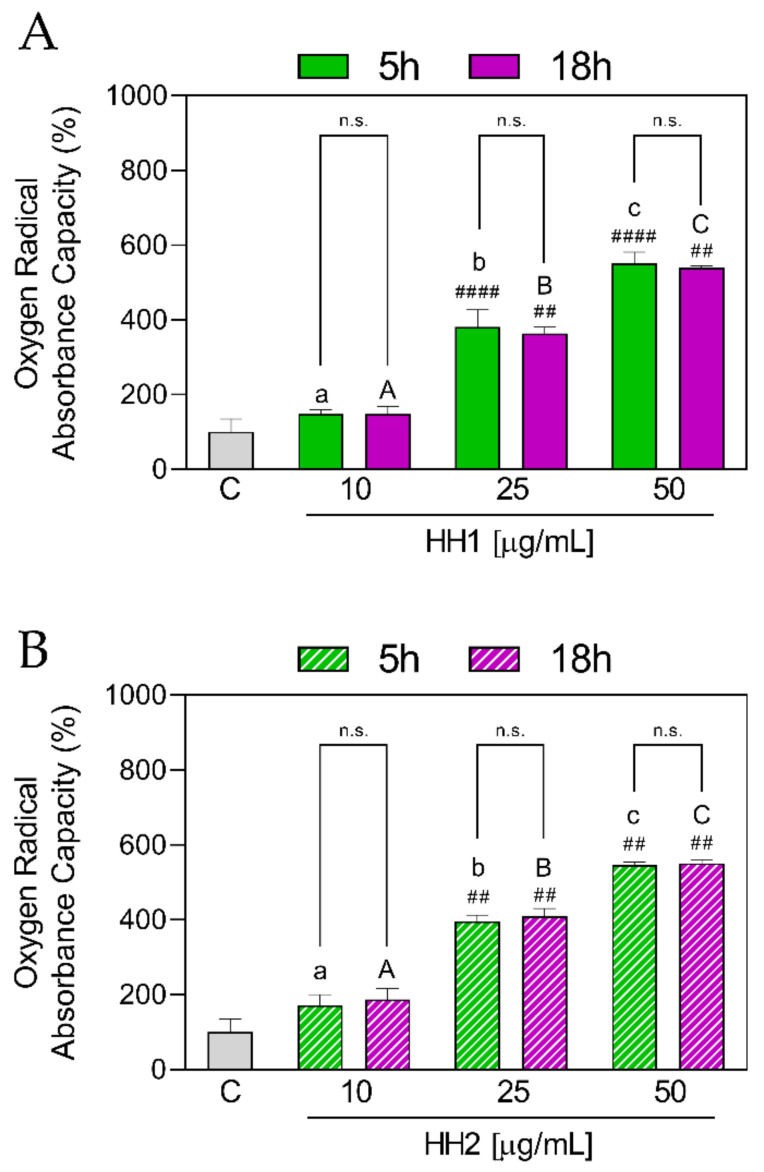
In vitro antioxidant power evaluation of the HHs by the oxygen radical absorbance capacity (ORAC) assay. C, control group; HH, hemp hydrolysate 1 (**A**) and 2 (**B**). Data were shown as the mean ± standard deviation (*n* = 10). Mixed-effects analysis with multiple comparisons was applied. ##, *p* < 0.01; ####, *p* < 0.0001, with respect to the C group. n.s., not significant. Different lower letters indicate the statistically significant difference (*p* ≤ 0.05) between the different concentrations of the HH obtained by 5 h of hydrolysis. Different upper letters indicate the statistically significant difference (*p* ≤ 0.05) between the different concentrations of the HH obtained by 18 h of hydrolysis.

The AAPH scavenging activity exerted by HH1 and HH2 was measured at different concentrations using the ORAC assay. As shown Figure 4A, HH1.5 statistically increased the ORAC values at 25 µg/mL (381 ± 45.50%, *p* < 0.0001) and 50 µg/mL (552 ± 28.90%, *p* < 0.0001) in comparison to the C group. In the same way, HH1.18 statistically increased the ORAC values at the same concentrations (25 µg/mL: 363 ± 17.2%, *p* = 0.032; 50 µg/mL: 539 ± 4.70%, *p* = 0.016). The increment was dose-dependent, finding significant differences between all the concentrations for HH1.5 and HH1.18, respectively (Figure 4A).

When the ORAC results in HH1.5 and HH1.18 were compared, no significant differences were found between both hydrolysates in any of the concentrations.

Regarding HH2, HH2.5 and HH2.18 were shown to increase ORAC levels at 25 (HH2.5: 396 ± 16.80%, *p* = 0.0021; HH2.18: 409 ± 20.70%, *p* = 0.0012) and 50 µg/mL (HH2.5: 547 ± 7.83%, *p* = 0.0014; HH2.18: 551 ± 10.00%, *p* = 0.0011) (Figure 4B). For each HH, there were significant differences between the three concentrations.

The comparison between HH2.5 and HH2.18 showed a significant difference at 10 µg/mL (*p* = 0.0012), not finding significant changes at 25 and 50 µg/mL (Figure 4B).

#### 3.1.5. HH1 and HH2 Show Differences in the Antioxidant Activity

After confirming the antioxidant activity of both hydrolysates (HH1 and HH2), they were compared with each other. As Table 2 showed, HH1.5 exerted a higher DPPH and TEAC radicals scavenging activity in comparison with HH2.5. However, no significant differences were found in the FRAP and ORAC values. On the other hand, when HH1.18 and HH2.18 were compared, HH2.18 showed a higher TEAC radicals scavenging activity at 25 µg/µL, as well as higher levels of AAPH radical scavenging at all concentrations. HH1.18 and HH2.18 did not show significant differences in the DPPH radical scavenging and FRAP values.

### 3.2. Analysis and Characterization of HHs Composition

**Figure 5:**
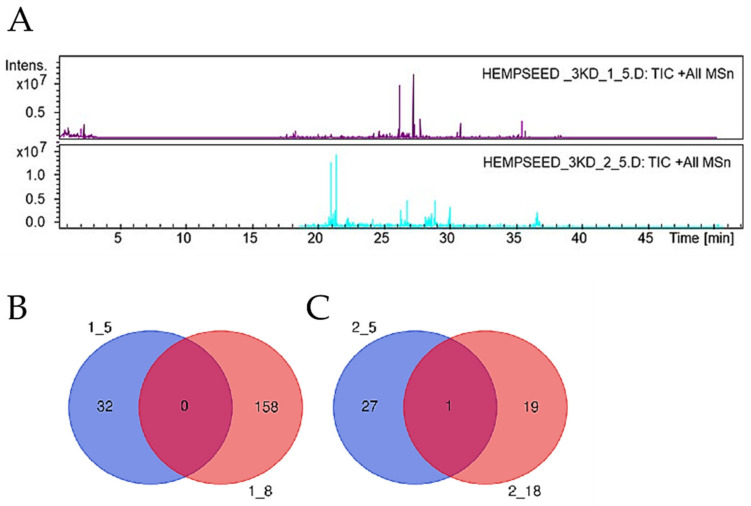
Analysis and characterization of HHs composition. (**A**) TIC of 3 kDa enriched peptides of HH1.5 and HH2.5. (**B**) Venn diagrams of the number of identified hempseed peptides between HH1.5 (blue circle) and HH1.18 (red circle). (**C**) Venn diagrams of the number of identified hempseed peptides between HH2.5 (blue circle) and HH2.18 (red circle).

HH1 and HH2 at 5 and 18 h of digestion, respectively, were characterized by LC-MS/MS analysis. Figure 5A shows the TIC obtained by LC-MS/MS analysis of 3 kDa peptides, respectively, for HH1.5 and HH2.5. Overall, the peptides belonging to Edestin2, acyl-activating enzyme 12, acyl-activating enzyme 15, acyl-activating enzyme 8, acyl-activating enzyme 4, and RNA polymerase beta subunit were identified in HH1, whereas, the peptides belonging to Edestin1, Edestin2, RNA polymerase beta’ subunit (chloroplast), hypothetical chloroplast RF21 (chloroplast), and photosystem I P700 apoprotein A2 (chloroplast) were detected in hempseed hydrolysate HH2 (Table S1). The different composition reflects the conditions of hydrolysis used for obtaining both hydrolysates. The conditions and enzymes used selectively produced two unique hydrolysates with a specific peptide composition. Moreover, our finding underlines the impact of the time of hydrolysis on the peptide composition of each hydrolysate. In fact, the hydrolysis performed for 18 h compared to 5 h produces a more prosperous complex of peptides, and no peptide has been found common to the two hydrolysates (Figure 5B). By analyzing the peptide sequences, we observed that some peptides during the 18 h of hydrolysis suffered hydrolytic cleavages, i.e., the (L)GPLMFGASKTLLNADHYDVY(F) form acyl-activating enzyme 12 detected in type 1 hydrolyzed after 5 h, has been hydrolyzed to (G)PLMFGASKTLL(N) after 18 h of digestions. A similar occurrence happened for the shorter peptides (L)NGASCIVFEGAPN(Y) from acyl-activating enzyme 15, which was generated by hydrolytic cutting from (Y)VTYGPLLNGASCIVFEGAP(N) and (G)PLLNGASCIVFEGAPNYPDSGRCWDIV(D) present in the hydrolysate HH1.5.

Similar behavior was observed for the HH2; only one peptide GVADWVYNNGDSPLVL, which belongs to Edestin2, was found to be present in both hydrolysates obtained after 5 and 18 h of hydrolysis (Figure 5C). These findings suggest the resistance of this peptide at the time of hydrolysis.

### 3.3. The HHs Reduce the H_2_O_2_-Induced Oxidative Stress in Caco-2 Cells

**Figure 6:**
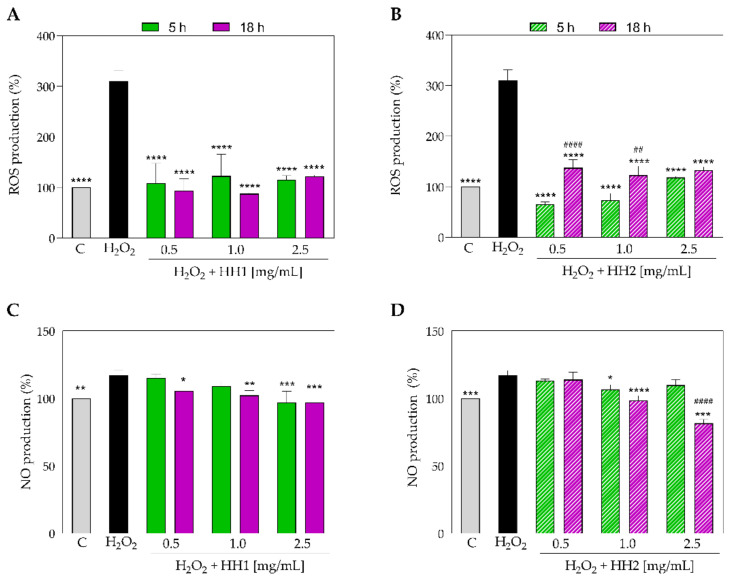
ROS (**A**,**B**) and NO (**C**,**D**) level production in H_2_O_2_-stimulated Caco-2 cells and treated with HH1.5/18 and HH2.5/18, respectively. Data were shown as the mean ± standard deviation (*n* = 4). Ordinary one-way ANOVA with multiple comparisons was applied. ##, *p* < 0.01; ####, *p* < 0.0001 with respect to the 5 h of hydrolysis. *, *p* ≤ 0.05; **, *p* < 0.01; ***, *p* < 0.001; ****, *p* < 0.0001, with respect to the H_2_O_2_ group. C group; HH, hemp hydrolysate.

To investigate the capacity of HHs (5 and 18 h) to modulate the ROS overproduction induced by H_2_O_2_, cellular experiments were performed. Our findings clearly demonstrated that Caco-2 cells treated with H_2_O_2_ alone showed an increase in the ROS levels up to 309.80 ± 21.02% versus the control cells. Figure 6A shows that HH1 (5 h and 18 h) reduced the intracellular H_2_O_2_-induced ROS levels up to 108.50 ± 39.23% and 93.64 ± 23.29% at 0.5 mg/mL, respectively. At 1 mg/mL, the reduction was up to 122.6 ± 42.84% and 87.56 ± 1.891%, respectively, while the same samples tested at 2.5 mg/mL decreased the ROS levels up to 115.2 ± 8.34% and 122.10 ± 2.41%, respectively.

Regarding the HH2 (5 and 18 h) samples, the results clearly showed that they were capable of reducing the H_2_O_2_-induced ROS overproduction up to 65.60 ± 4.06% and 137.30 ± 16.01%, at 0.5 mg/mL, respectively; up to 73.42 ± 13.48% and 122.70 ± 17.79% at 1.0 mg/mL, respectively; and up to 118.50 ± 1,75% and 132.60 ± 6,58% at 2.5 mg/mL, respectively (Figure 6B).

The effects of HHs (5 and 18 h) on the production of NO levels were evaluated on human intestinal Caco-2 cells after oxidative stress induction. Notably, H_2_O_2_ (1 mM) treatment induced an oxidative stress that led to an increase in the intracellular NO levels up to 117.00 ± 3.64% (Figure 6C,D). Pre-treatment with HH1 (5 h and 18 h) decreased the H_2_O_2_-induced NO overproduction up to 115.20 ± 2.80% and 105.40 ± 1.55% at 0.5 mg/mL, respectively; up to 109.00 ± 3.56% and 102.20 ± 3.56% at 1.0 mg/mL, respectively; and up to 96.86 ± 8.40% and 96.86 ± 6.16, at 2.5 mg/mL, respectively (Figure 6C). On the other hand, the pre-treatment with HH2 (5 and 18 h) decreased the H_2_O_2_-induced NO overproduction up to 113.00 ± 1.34% and 113.90 ± 5.44%, at 0.5 mg/mL, respectively; up to 106.70 ± 3.39% and 98.65 ± 3.39% at 1.0 mg/mL, respectively; and up to 109.90 ± 4.11% and 81.61± 2.80% at 2.5 mg/mL, respectively (Figure 6D).

### 3.4. Trans-Epithelial Transported HHs Exert Antioxidant Activity

**Figure 7:**
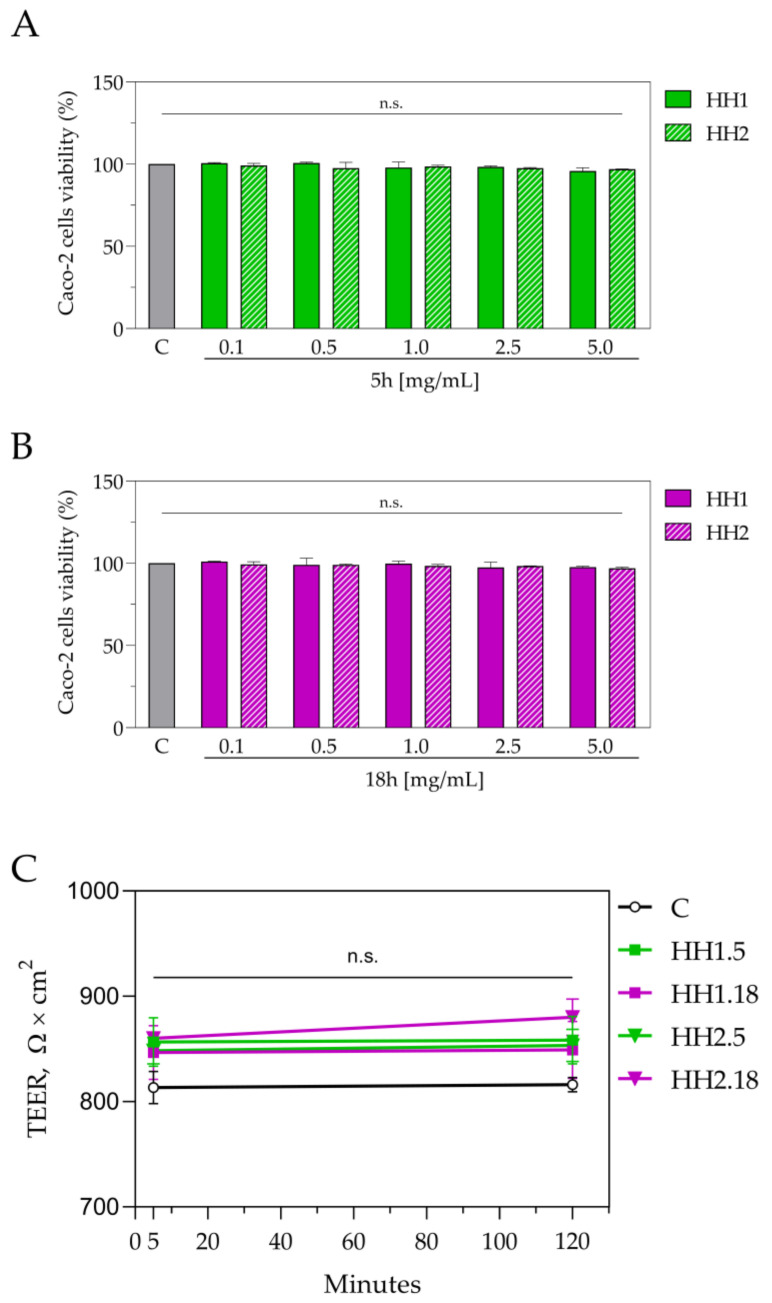
Effect of HHs on the Caco-2 viability and cellular monolayer integrity and permeability assessed by MTT (**A**,**B**) and TEER measurement (**C**). Data were shown as the mean ± standard deviation (*n* = 4). Ordinary one-way ANOVA with multiple comparisons was applied. C: control group; n.s., not significant; TEER, trans-epithelial electrical resistance.

Preliminary MTT experiments were performed on Caco-2 cells before the evaluation of the HHs trans-epithelial transport. The results suggested that HHs are safe for human intestinal Caco-2 cells. Indeed, no effects on cellular viability were observed (Figure 7A,B). Hence, differentiated Caco-2 cells were separately incubated with both HH1.5/18 and H2.1/18 h in the apical (AP) chamber of the trans-well system, and after 2 h, the basolateral solutions (BL) were collected and analyzed. TEER measurements at the beginning and at the end (2 h) of the experiments suggested that both the HH1.5/18 and H2.1/18 h samples did not affect the cellular monolayer’s integrity and permeability (Figure 7C). Notably, each recovered BL solution was tested for assessing both its chemical composition and its ability to maintain its antioxidant capacity. In this context, the list of the trans-epithelial transported peptides is reported in Table S2, whereas Table 3 shows the peptide fragments identified in BL medium versus the peptide precursors identified in HH1.5 and H1.18 hydrolysates, respectively.

**Figure 8:**
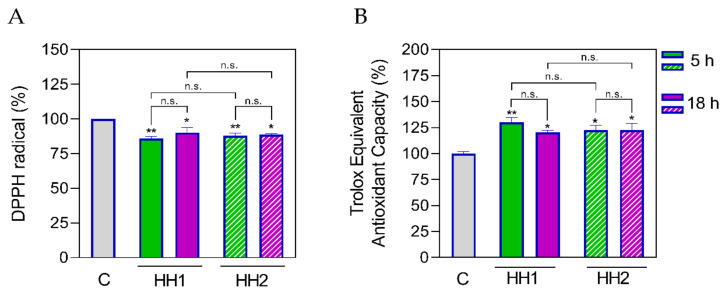
Confirmation of the antioxidant effect of HHs (1 mg/mL) after the trans-epithelial transport by differentiated Caco-2 cells, measured by DPPH (**A**) and ABTS (**B**) assays, respectively. Data were shown as the mean ± standard deviation (*n* = 4). Ordinary one-way ANOVA with multiple comparisons was applied. *, *p* ≤ 0.05; **, *p* < 0.01, with respect to the C: control group; n.s., not significant.

In addition, each recovered BL solution was contemporarily assessed for evaluating the antioxidant effects exerted by HHs; thus, both DPPH and TEAC assays were performed. As shown in Figure 8A both HH1.5 and HH1.18 reduced the DPPH radical by 14 (*p* = 0.005) and 9.80% (*p* = 0.02), respectively. In addition, both HH1.5 and HH1.18 increased the TEAC values with respect to the C untreated group (HH1.5: 130 ± 4.53%; *p* = 0.0044, HH1.18: 121 ± 1.84%, *p* = 0.0232) (Figure 8B). No significant differences were found between HH1.5 and HH1.18 in any of the trials. Regarding HH2, HH2.5 and HH2.18 also reduced the DPPH radical (HH2.5: 87.80 ± 1.76%, *p* = 0.008; HH2.18: 88.70 ± 0.69, *p* = 0.012) in comparison to the C group. In the same way, both HHs increased the TEAC (HH2.5: 122 ± 4.53%, *p* = 0.01; HH2.18: 122 ± 6.36%, *p* = 0.01). When HH2.5 and HH2.18 were compared between them, no significant differences were found. Finally, the antioxidant effects of HH1 and HH2 were compared. HH1.5 and HH2.5 did not show significant differences (*p* > 0.05). The same situation was observed for HH1.18 and HH2.18.

## 4. Discussion

Food-derived peptides have gained the attention of the scientific community because of their several beneficial health effects [2,17]. These pleiotropic effects have been demonstrated to be linked mainly to the physicochemical composition of the peptides, which in turn, is related to the conditions in which the hydrolysis process occurs [13,35,36]. This hydrolysis can be carried out by chemical or biological processes. Within the biological process, enzymatic hydrolysis through the use of natural or commercial proteases is the most widely used technique due to its numerous advantages. The conditions under which the hydrolysis process is carried out (temperature, pH, time, and enzyme used) are deeply optimized [1,37,38]. In the present study, the antioxidant effect of four hempseed protein hydrolysates (HHs), obtained with different enzymes and time of hydrolysis, was demonstrated by combining several antioxidant tests (DPPH, TEAC, FRAP, and ORAC assays, respectively).

Oxidative stress has been proposed as a key molecular process in the onset and development of multiple chronic diseases [39]. For this reason, the search for antioxidant compounds to be used for the therapy of these diseases is of great interest in the current fields. In this aspect, the pharmaceutical and food industries have shown interest in the development/production of nutraceuticals and functional foods with antioxidant activity [40]. The results of the present work showed that four HHs obtained after different hydrolysis conditions have antioxidant effects that are moreover higher in comparison with other HHs obtained using different hydrolytic enzymes, i.e., pepsin (P) and pancreatin (Pa), Alcalase (A), A and Flavourzyme, or Protamex [19,25,27,28,29]. Specifically, previous studies have demonstrated a reduction in the DPPH radical by 5% in HH (1 mg/mL) obtained with P and Pa hydrolysis [19]. We observed that the percentage of the DPPH’s radical scavenging activity was ~60% for HH1.5 at 1 mg/mL. Furthermore, two hydrolysates, obtained after Alcalase and Alcalase and Flavourzyme hydrolysis of hempseed, showed a percentage of DPPH radical scavenging activity of 40% at a concentration of 10 mg/mL [29], suggesting that this peptide mixture is 10 times less active than the ones investigated in this work. On the other hand, our results clearly indicate that HH samples display a higher percentage of TEAC and FRAP levels (in the tested range of concentration of 10, 25, and 50 µg/mL) compared to hempseed hydrolysates produced with Protamex, which increased the TEAC and FRAP levels by 50% at 0.4 mg/mL [28].

As indicated, other hempseed hydrolysates and single isolated peptides have demonstrated that they can exert antioxidant effects in cell-free systems [10,30,41], in cell-based conditions on HepG2 [26,28], HaCaT, and HeLa cell lines [30], and in in vivo systems (spontaneously hypertensive rats) [27]. However, the enzymes used to obtain the hydrolysate in these studies were Alcalase, Flavoyrzyme, Neutrase, Protamex, pepsin, trypsin, and pancreatin. To date, no studies have identified the antioxidant effect of peptides obtained after the use of digestive enzymes from *Aspergillus niger*, *Aspergillus oryzae*, or *Bacillus licheniformis*.

Our findings clearly indicated that the hydrolysate obtained after 5 h presents a higher antioxidant activity compared to the hydrolysate obtained after the hydrolysis period of 18 h. This is probably due to the fact that 5 h of hydrolysis are sufficient to obtain bioactive peptides of small/medium size with higher antioxidant activity. A drastically time-extensive hydrolysis (18 h) can mainly generate small-sized peptides, dipeptides, and tripeptides that have previously shown a lower antioxidant capacity compared to tetrapeptides and pentapeptides [25,27]. Five hours of hydrolysis rather than 18 h implies an improvement in the energy level, improving its performance, and generating benefits in terms of costs and energy savings, which would be framed within a sustainable industrial process.

In addition, within 5 h, HH1, obtained after the hydrolysis with the cocktail of enzymes from *Aspergillus niger* and *Aspergillus oryzae* (pectin lyase, polygalacturonase, cellulase, oryzin, and leucyl aminopeptidase), showed higher levels of antioxidant capacity than HH2, obtained after the hydrolysis with enzymes from *Aspergillus niger* and *Bacillus licheniformis*. This result can be explained considering that co-digestion for a shorter time (5 h) is more efficient in generating antioxidant peptide species. In this sense, oryzin and Leucyl aminopeptidase are used in HH1 obtention but not in HH2, while subtilisin is only used for HH2.

Although food-derived bioactive peptides have shown numerous positive health effects, some factors limit their commercialization, including their bitter taste, hygroscopicity, and low bioavailability [17,42]. As is well known, after ingestion, the structure and therefore the bioactivity of peptides can change during the processes of digestion, absorption, and transport through the intestinal epithelial cells toward the blood circulatory system. In this regard, it has been shown that the bioactivity of peptides can be maintained, decreased or lost, and increased or changed [43,44]. Of all the studies that have shown the antioxidant activities of peptides and hydrolysates derived from hempseed, only the study by Bollati et al. demonstrated that five peptides (DVFSPQAGRL, WVSPLAGRT, IGFLIIWV, DVFTPQAGRIST, and IRALPEAV), identified in a hempseed peptide mixture obtained with pepsin, were able to cross the Caco-2 cell barrier and maintain their antioxidant effects [26]. Moreover, WVSPLAGRT and IGFLIIWV were also demonstrated to possess anti-inflammatory activities in HepG2 cells [24].

In the present study, the antioxidant activity of the four hydrolysates was maintained after the safe trans-epithelial transportation through Caco-2 cells. Specifically, both hydrolysates decreased the production of ROS and NO in Caco-2 cells stimulated with H_2_O_2_. Our results are totally in agreement with previous studies that have demonstrated that other hempseed hydrolysates and peptides decreased ROS production in Caco-2, HepG2, HaCaT, HeLa, and HUVEC cell lines [26,28,30,31].

On the other hand, as reported in Figure 7, the HHs samples did not affect Caco-2 viability and cellular monolayer integrity or permeability. In addition, the peptidomic profile of the transported peptides is different compared to the one from the original mixtures (Table 3). This result can be explained considering that the differentiated Caco-2 cells are polarized cells that, on the brush border level (AP side), express different peptidases that actively modulate the peptidomic profile of the peptide mixture, leading to the formation of break-down fragments that are newly generated and are selectively transported in the BL solution.

Thus, by comparing the composition of each absorbed peptide in HH1 and HH2, obtained after 5 and 18 h, respectively, HH1.5 is the richest hydrolysate in terms of the absorbed peptides. At the same time, smaller peptides that were not detected in the original hydrolysates, i.e., QSFILG, ASVTKLG, ASVTKLG, KGVEIEG, VIEGKEG, GKNKTAI, QDVNSLG, NLDVSKG, GKKISESC, GKNKTAI, IGMKFG, KGVEIEG, and QEDNEKHI with low molecular weight < 1 kDa belonging to *Cannabis sativa* proteins, but not present in HH1.5 hydrolysate itself, were identified. This result can be explained considering that the differentiated Caco-2 cells can act as a natural and active sieve of new peptide species that are selectively transported by the cells concentrating their presence in the BL solution and therefore becoming detectable since their abundance is easily detectable by LC-MS. By acting as a natural sieve, Caco-2 cells are able to reduce the complexity of the peptide mixture, contributing to the detection of peptides that despite being present in the HH1.5 hydrolysate, were not abundant enough to be detected.

It is known that there are four different transport pathways for peptides to cross epithelial cells into the bloodstream: (1) the PepT1 transporter, (2) the paracellular pathway through tight junctions, (3) transcytosis, and/or (4) passive transcellular diffusion [43,45]. Working with protein hydrolysates, in which many bioactive peptides exist, it is reasonable to consider that more than one of these mechanisms may occur; therefore, it is not feasible to characterize the mechanism of action through which peptide mixtures cross the differentiated human intestinal Caco-2 cells. However, overall, it was clearly demonstrated that peptides within HHs are capable of passing the gastrointestinal barrier and are still able to exert antioxidant capacity. This knowledge is of great importance for the future commercialization of these HHs.

## 5. Conclusions

The results of this work demonstrated that four HHs exert an antioxidant effect in vitro and maintain this effect after crossing the transepithelial barrier, as mimicked in vitro employing differentiated Caco-2 cells. Due to the importance of oxidative stress in the development of the main chronic diseases, these could be used in the future as ingredients in the generation of functional foods or nutraceuticals for the prevention and treatment of chronic diseases. This is the first study to show the antioxidant effect of HHs after crossing the in vitro gastrointestinal barrier. However, this sheds light on future in vivo studies on the effects of HHs on oxidative stress-derived diseases. In addition, these results provide a clear indication for the industrial exploitation of these HHs, suggesting that, to obtain an active antioxidant peptide mixture, fewer time-extensive hydrolytic processes in combination with a co-digestion using well-selected enzymes should be realized.

## Figures and Tables

**Table 1:** Enzymes used to generate the HHs.

Source	EC Number	Family	Enzyme	HH1	HH2
*Aspergillus niger*	4.2.2.10	Polysaccharidases	Pectin lyase	✓	✓
	3.2.1.15	Glycosidases	Polygalacturonase	✓	✓
	3.2.1.4		Cellulase	✓	✓
*Aspergillus oryzae*	3.4.21.63	Peptidase S8	Oryzin	✓	
	3.4.11.1	Peptidase M17	Leucyl aminopeptidase	✓	
*Bacillus licheniformis*	3.4.21.62	Peptidase S8	Subtilisin		✓

**Table 2:** Panel of antioxidant activity of HH1 and HH2.

Assay	Concentration	5 h		*p*-Value	18 h		*p*-Value
		HH1	HH2		HH1	HH2	
DPPH	0.5 mg/mL	54.50 ± 3.43	83.80 ± 5.96	0.005	82.70 ± 0.96	83.10 ± 4.52	>0.999
	1.0 mg/mL	40.10 ± 2.08	62.70 ± 2.06	0.008	58.80 ± 4.07	60.90 ± 3.16	0.961
	2.5 mg/mL	68.00 ± 6.37	40.00 ± 1.22	0.047	41.00 ± 0.89	35.70 ± 1.25	0.088
TEAC	10 µg/mL	133 ± 5.70	111 ± 9.69	0.009	107 ± 12.30	117 ± 8.13	0.183
	25 µg/mL	169 ± 11.80	148 ± 3.50	0.021	145 ± 4.61	154 ± 3.74	0.044
	50 µg/mL	205 ± 5.28	177 ± 6.93	0.0001	174 ± 5.56	175 ± 12.7	>0.999
FRAP	10 µg/mL	235 ± 60.70	192 ± 93.00	0.785	196 ± 94.90	216 ± 86.60	0.999
	25 µg/mL	646 ± 36.80	589 ± 37.30	0.376	566 ± 73.40	575 ± 69.60	>0.999
	50 µg/mL	1578 ± 39.00	1397 ± 150.00	0.141	1186 ± 25.90	1392 ± 195.00	0.209
ORAC	10 µg/mL	148 ± 12.00	172 ± 27.60	0.100	148 ± 20.00	187 ± 28.70	0.001
	25 µg/mL	381 ± 45.50	396 ± 16.80	0.997	363 ± 17.20	409 ± 20.7	0.001
	50 µg/mL	552 ± 28.90	547 ± 7.83	>0.999	539 ± 4.70	551 ± 10.00	0.103

DPPH, 1-diphenyl-2-picrylhydrazyl radical; TEAC, Trolox equivalent antioxidant capacity; FRAP, ferric reducing antioxidant power; ORAC, oxygen radical absorbance capacity; HH, hemp hydrolysate. Data were shown as the mean ± standard deviation (*n* = 10).

**Table 3:** Trans-epithelial transported peptides identified in BL medium. Sequences are described with the 1-letter amino acid code. HH1.5, hydrolysate 1 obtained after 5 h of hydrolysis; HH1.18, hydrolysate 1 obtained after 18 h of hydrolysis. BL refers to the basolateral portion.

Protein	Sequence	*m*/*z* (Da)	Sample
HH1.5			
acyl-activating enzyme 12	(H)AHGRADDTMNLGGIKVSSVE(I)	686.43	1.5
	(Y)HAHGRADDT(M)	327.32	1.5BL
acyl-activating enzyme 8	(D)GGHKPGSVGKPVGQEMAILDQ(N)	702.54	1.5
	(G)HKPGSVGKPVGQEMAILDQ(N)	664.56	1.5BL
HH1.18			
RNA polymerase beta subunit	(L)GVPSRMNVGQIFECSLGLAGEL(L)	759.61	1.18
	(D)MVFNPLGVPSRMNVGQIFECSL(G)	813.24	1.18
	(V)PSRMNVGQIFE(C)	426.74	1.18BL
ATP synthase CF1 alpha subunit (chloroplast)	(V)LMGDGLLIQEGSSVKATGRIAQIPVSEAFL(G)	1034.28	1.18
	(A)TGRIAQIPVSEAF(L)	463.69	1.18BL
ATP synthase CF1 beta subunit (chloroplast)	(G)LMRGMDVIDTGAPLSVPVGGATLGRIF(N)	914.98	1.18
	(P)LSVPVGGATLGRI(F)	620.66	1.18BL

## Data Availability

The data presented in this study are available in the article and Supplementary Materials.

## Supplementary Materials

The following supporting information can be downloaded at: https://www.mdpi.com/article/10.3390/antiox12051099/s1, Table S1: list of peptides and proteins from which they belong; Table S2: list of trans-epithelial transported peptides.
